# The Research Progress on the Effects of Phytohormones on Nitrogen Use Efficiency in Rice

**DOI:** 10.3390/plants14142193

**Published:** 2025-07-15

**Authors:** Kunlun Liu, Xingyi Liang, Weiling Wang, Zhongyang Huo, Can Zhao

**Affiliations:** Jiangsu Key Laboratory of Crop Genetics and Physiology, Jiangsu Key Laboratory of Crop Cultivation and Physiology, Jiangsu Co-Innovation Center for Modern Production Technology of Grain Crops, Agricultural College, Yangzhou University, 88 Daxue South Road, Yangzhou 225009, China; liunan990523@gmail.com (K.L.); 18251378057@163.com (X.L.); weilingw@163.com (W.W.); huozy69@163.com (Z.H.)

**Keywords:** Hormone Signaling, nitrogen uptake, *Oryza sativa*, sustainable agriculture

## Abstract

Nitrogen (N) is one of the most important nutrients determining crop growth performance. With the increasing demand for sustainability in global agriculture, improving nitrogen use efficiency in rice has become a critical issue. Nitrogen use efficiency (NUE) in rice is a complex trait influenced by multiple factors, among which phytohormones play a key role. NUE is primarily regulated through the influence of phytohormones on absorption, transport, assimilation, and utilization processes. In this review, we focus on these interactions and summarize the relationships between major hormones and nitrogen use efficiency in rice. Finally, we outline the current challenges and future research prospects in this field. Although studies have shown promising results for their role in improving crop NUE, the specific mechanisms remain unclear. Additionally, the interactions among phytohormones and the influence of environmental factors on their effectiveness require further investigation. This review provides theoretical support and technical guidance for understanding the role of phytohormones in rice NUE and offers insights into improving NUE in rice.

## 1. Introduction

Rice (*Oryza sativa* L.) is one of the most important staple crops in the world, primarily grown in Asia and accounting for 90% of global production [1]. As the largest producer and consumer of rice, China has a total rice-growing area of nearly 30 million hectares, with production exceeding 212 million tons in 2018, about 27% of global rice production [1]. It is estimated that by 2030, rice production will need to increase by approximately 20% to meet China’s growing demand [1,2]. Low agricultural productivity and excessive fertilizer use are common issues in China and many developing countries, leading to severe environmental pollution and high environmental management costs [3]. Currently, rice production in China is on a positive trajectory, with a slight expansion in planting area, continuous improvements in yield, and better grain quality [4]. Total production has remained stable at over 200 million tons for the past 10 years. Domestic rice stocks are sufficient, consumption is steadily increasing, and the stock-to-consumption ratio far exceeds the global average [4]. Despite the central role of nitrogen in rice production, its excessive use and low uptake efficiency have caused serious environmental and economic concerns. Understanding how phytohormones influence nitrogen metabolism remains an open question, making this a critical area for targeted research.

Phytohormones are naturally occurring small-molecule compounds present in trace amounts in plants [5]. Their highly interconnected signaling pathways allow plants to finely tune growth and development in response to environmental cues, including growth conditions, nutrient use efficiency, and both biotic and abiotic stresses [6]. To better understand how phytohormones influence nitrogen use efficiency (NUE) in rice, the following section first introduces the classification, chemical structures, and physiological functions of major phytohormones. This will provide a theoretical foundation for examining their regulatory roles in nitrogen uptake and utilization. Phytohormones are classified into several categories based on their structure and physiological function. The most common categories include auxins, cytokinins (CKs), abscisic acid (ABA), gibberellins (GAs), brassinosteroids (BRs), salicylic acid (SA), jasmonic acid (JA), ethylene (ET), and newly discovered phytohormones such as strigolactones (SLs) [7]. Each category has distinct roles and can regulate plant growth through synergistic or antagonistic interactions with other phytohormones [8,9]. Phytohormones are closely linked to rice yield formation, but their content in rice tissues is very low, making extraction difficult and costly. Therefore, synthetic phytohormones (plant growth regulators) are generally used in agricultural production, typically produced via microbial fermentation or chemical synthesis [10]. Insufficient photosynthetic capacity is a major factor limiting yield improvements in rice. Hormone treatments can enhance the photosynthetic capacity of leaves and the transport of photosynthates to grains, resulting in a significant increase in per-plant yield [11].

Nitrogen is the mineral nutrient required in the largest amount for crop production, and 40–60% of the increase in global food production can be attributed to fertilizer use, highlighting the critical importance of nitrogen fertilizers for global food security [12]. Nitrogen use efficiency (NUE) is defined as the yield (in grain or biomass) obtained per unit of available nitrogen [13]. NUE covers the entire process of nitrogen absorption, assimilation, and utilization by plants [14]. Plants primarily absorb nitrogen from the soil in two inorganic forms, nitrate (NO_3_^−^) and ammonium (NH_4_^+^), through nitrate transporters (NRTs) and ammonium transporters (AMTs), respectively. Absorbed nitrate is first reduced to nitrite by nitrate reductase (NR); then, nitrite is reduced to ammonium by nitrite reductase (NiR) in plastids and/or chloroplasts. Ammonium is assimilated into amino acids via the glutamine synthetase (GS)–glutamate synthase (GOGAT) cycle. The genes responsible for N uptake and N assimilation are mainly *OsNRT1.1a*, *OsNRT1.1b*, *OsNPF2.4*, *OsNPF3.1*, *OsAMT1;1*, *OsAMT1;2*, *OsAMT1;3*, *OsAMT2;1*, and *OsAMT3;1* [15,16]. The average nitrogen fertilizer use efficiency in China is only about 28.3–35.0%, significantly lower than the global average of 40.0–60.0% [17,18]. Low nitrogen use efficiency not only limits rice yield and causes fertilizer waste, but also harms the environment. Effectively improving nitrogen fertilizer efficiency to increase yield is crucial for ensuring food security in China [19,20]. Previous research has extensively explored the timing and rate of nitrogen fertilizer application, but studies on the effects of phytohormones and plant growth regulators on nitrogen uptake and utilization efficiency are relatively limited. This review aims to summarize the effects of several phytohormones on rice NUE, particularly focusing on the interactions between phytohormones and nitrogen uptake and utilization, in order to provide a reference for further exploration of how phytohormones affect nitrogen uptake and utilization in rice.

## 2. Phytohormones

### 2.1. Auxins

Auxins have been extensively studied for their role in regulating various aspects of plant growth and development, such as embryo development, cell elongation, cell division, apical dominance, lateral root formation, responses to light and gravity, nutrient absorption, and vascular tissue differentiation [21,22](Table 1). IAA is mainly synthesized via the tryptophan-dependent indole-3-pyruvic acid (IPA) pathway. In this pathway, tryptophan is first converted to IPA by TRYPTOPHAN AMINOTRANSFERASE OF ARABIDOPSIS1 (TAA1) and its homologs, and then IPA is subsequently converted to IAA by the flavin-containing monooxygenase YUCCA (YUC) family [23,24]. Other minor pathways include the indole-3-acetamide (IAM) and indole-3-acetaldehyde (IAAld) routes, but the TAA/YUC pathway is considered the predominant biosynthetic route in rice and many other plant species.

Auxin has been extensively reported to modulate nitrogen uptake and utilization in rice. Under low-nitrogen conditions, exogenous application of low concentrations of indole-3-acetic acid (IAA) significantly enhances nitrogen uptake and promotes root development in rice seedlings [25,26]. Conversely, reduced auxin sensitivity leads to abnormal root responses to nitrogen supply. For example, the dgt mutant in tomato, which is defective in auxin signaling, exhibits greater tolerance to nitrogen deficiency, suggesting that auxin pathways are finely tuned under nitrogen stress [26]. Li et al. (2024) proposed that auxin may serve as a central regulatory hub for enhancing nitrogen use efficiency (NUE) in crops, but emphasized that its effects are highly context-dependent, varying with plant species, nitrogen form, and developmental stage [22]. This underscores the need for more rigorous investigation into auxin–nitrogen interaction mechanisms, particularly under field conditions. Saito et al. [27] reported that the transcriptional repressor IAA17 in Arabidopsis modulates ammonium assimilation and overall nitrogen status by regulating the expression of the cytosolic glutamine synthetase gene *GLN1;2*. In rice, auxin similarly influences nitrogen use efficiency (NUE) by modulating the expression of genes involved in nitrogen metabolism. De Jong et al. [28] demonstrated that nitrogen deficiency-induced tillering involves a regulatory interaction between auxin and strigolactones. Under high-ammonium conditions, repression of auxin biosynthesis reduces shoot apical meristem activity. Ammonium toxicity, a major constraint in rice cultivation, is closely linked to dynamic changes in auxin levels. High concentrations of NH_4_^+^ suppress auxin biosynthesis in roots, leading to reduced free IAA levels and impaired root development [29]. The ability to maintain elevated auxin levels in roots is critical for ammonium tolerance, as NH_4_^+^ is a primary nitrogen source in rice cultivation. Modulating auxin biosynthesis may thus enhance ammonium tolerance and ultimately reshape nitrogen uptake and utilization efficiency in rice. Auxin influences nitrogen use efficiency (NUE) by reshaping root system architecture. Under low-nitrogen conditions, plants exhibit elevated root auxin levels compared to those grown under high nitrogen. This suggests a conserved mechanism shared by both dicots and monocots, in which root auxin levels are modulated in response to the plant’s nitrogen status [30]. Notably, this auxin-mediated branching process requires ammonium-induced apoplastic acidification, which is dependent on AMT-type ammonium transporters that facilitate auxin transmembrane movement. AMT-dependent acidification is a prerequisite for enhancing auxin mobility and triggering lateral root formation [31]. In addition, the dual-affinity nitrate transporter and sensor NRT1.1 (also known as NPF6.3) couples nitrate signaling with auxin transport, integrating external nitrogen cues into root developmental responses. Bouguyon et al. [32] found that under low nitrate availability, NRT1.1 actively transports auxin away from developing root tips, thereby suppressing lateral root initiation. This nitrate–auxin interaction mechanism fine-tunes the plasticity of root system architecture in response to external nitrogen levels. Although many mechanistic insights have been gained from hydroponic and controlled-environment studies, their applicability in agronomic practice remains uncertain. Hu et al. (2024) [33] provided crucial field-based evidence demonstrating that localized nitrogen supply enhances rhizosphere nitrogen distribution and promotes root development, thereby improving rice yield and nitrogen use efficiency. Thus, auxin not only plays a central role in growth and development regulation but also demonstrates increasingly recognized functions in nitrogen uptake, root system architecture, and enhancing nitrogen use efficiency (NUE) in rice. Future strategies aimed at manipulating IAA biosynthesis, transport, or signaling components hold promise as key approaches to improve NUE.

### 2.2. Cytokinins

Cytokinins (CKs) exert important regulatory effects on plant morphology, physiology, and yield, and are among the main factors regulating nitrogen absorption, transport, and metabolism (Table 1). As key phytohormones in plant growth and development, cytokinins play vital roles in processes such as embryo development, yield formation, and stress resistance [34]. CKs are synthesized from adenine nucleotide precursors (AMP, ADP, or ATP), with the key enzyme being isopentenyltransferase (IPT). IPT catalyzes the transfer of an isopentenyl group from dimethylallyl diphosphate (DMAPP) to adenine nucleotides, forming isopentenyladenine-type nucleotide precursors such as iPRTP, iPDP, and iPMP. In rice, *OsIPT* genes primarily regulate the biosynthesis of isopentenyladenine-type (iP-type) and trans-zeatin-type (tZ-type) CK precursors. These iP-type nucleotide precursors are then hydroxylated by CYP735A family enzymes to generate tZ-type nucleotides. Finally, the nucleotide precursors are hydrolyzed by phosphatases or nucleotidases to release biologically active free-base CKs, such as tZ and iP [35,36].

Nitrogen availability directly affects cytokinin (CK) biosynthesis and distribution: high concentrations of nitrate (NO_3_^−^) stimulate CK synthesis in roots, which in turn inhibits root elongation by reducing meristematic activity [37,38]. Xu et al. [37] found that nitrogen-induced expression of *OsIPT* genes increases cytokinin levels in both roots and shoots, promoting rice tillering by suppressing FC1, a key downstream regulator in the strigolactone signaling pathway. The main product of nitrogen assimilation, glutamine, can upregulate the expression of *OsIPT* genes, thereby promoting the biosynthesis of trans-zeatin (tZ) and its riboside form (tZR) [39,40]. These cytokinin molecules participate in systemic nitrogen signaling, regulating nitrogen allocation between roots and shoots [40,41]. Exogenous application of cytokinins can enhance photosynthetic capacity, nitrogen uptake, and root physiological traits during the grain-filling stage in rice, thereby synergistically improving grain yield and nitrogen use efficiency (NUE) [34,41]. Shah et al. (2023) [41] further demonstrated under field conditions that foliar application of cytokinins (CKs) enhances grain yield and nitrogen use efficiency by promoting nitrogen-assimilating enzyme activities and facilitating nitrogen transport to the shoots. Increasing evidence suggests that under nitrogen stress conditions, cytokinin (CK) and abscisic acid (ABA) exhibit antagonistic interactions. CKs typically promote shoot growth and tillering, whereas ABA mediates stress responses and suppresses axillary bud outgrowth. This hormonal antagonism is finely regulated by nitrogen availability: Under high-nitrate conditions, CK biosynthesis and signaling are enhanced, leading to the repression of ABA-responsive genes such as *OsbZIP23* and *OsSAPK9*. In contrast, nitrogen deficiency or ammonium stress elevates ABA levels, which antagonize CK-induced meristematic activity, thereby suppressing tillering and altering root development in rice [42,43]. The crosstalk between CK and ABA signaling pathways thus plays a critical role in orchestrating rice growth strategies in response to nitrogen availability. However, excessive accumulation of cytokinins inhibits root elongation and diminishes nutrient acquisition capacity, indicating a trade-off between shoot development and root expansion under high-nitrogen conditions [38,44]. Local cytokinin biosynthesis is an important response to nitrogen. Nitrogen fertilizer promoted the synthesis of local cytokinins in rice, thus increasing the number of flowers in rice panicles [45]. Overexpression of *OsGRX6* gene could increase the content of cytokinin, affect several transporters in plants, and also change the nutritional status of rice plants so that the total nitrogen content in plants and seeds increased significantly [44]. However, cytokinins inhibited root elongation by negatively regulating root apical meristem activity, and the ubiquitin ligase EL5 helped maintain root meristem viability by increasing cytokinin levels [38]. A rapid supply of inorganic nitrogen elevated glutamine levels and induced the biosynthesis of novel cytokinins via *OsIPT4* [40]. The accumulated cytokinins inhibited *OsFC1* expression, thereby promoting axillary bud growth. Krouk et al. [46] found that supplying auxin and cytokinins prior to excision could maintain the mRNA, protein, and activity levels of nitrate reductase. Before cytokinin treatment, when roots were cultured in NO_3_^−^ medium, the transcriptional activation of nitrate reductase was delayed. This suggests that nitrate reductase controls the interaction between the NO_3_^−^ and cytokinin signaling pathways. Cytokinins can be synthesized de novo using nitrate (the primary inorganic nitrogen source) and glutamine (a key nitrogen assimilation product) as substrates [46].

Therefore, although optimizing cytokinin levels through genetic engineering or targeted cultivation management is an effective strategy to improve NUE, balanced regulation is necessary to avoid negative impacts on root growth or stress tolerance.

### 2.3. Abscisic Acid

Abscisic acid (ABA) is often called the plant “stress hormone” as it is involved in responses to both biotic and abiotic stresses (Table 1). Its precursor, farnesyl pyrophosphate (FPP), is synthesized from acetyl-CoA and influences many physiological and developmental processes, including seed maturation, dormancy, germination, and stress responses. ABA is synthesized from carotenoid precursors via an indirect biosynthetic pathway. The key step involves the cleavage of 9-cis-epoxycarotenoids by 9-cis-epoxycarotenoid dioxygenase (NCED), producing xanthoxin. Xanthoxin is then oxidized to abscisic aldehyde by ABA2, a short-chain dehydrogenase/reductase (SDR). Finally, abscisic aldehyde is further oxidized to ABA by AAO3 (abscisic aldehyde oxidase), a molybdenum cofactor (MoCo)-dependent enzyme [47].

Abscisic acid (ABA) also plays an important role in nitrogen uptake and utilization in rice by promoting root hair elongation, thereby enhancing nitrogen acquisition under nitrogen-deficient conditions [48]. However, its effects are highly environment-dependent. For example, nitrogen fertilization reduces endogenous abscisic acid (ABA) levels in leaves and increases the proportion of auxin, cytokinin, and gibberellin through hormonal rebalancing, thereby enhancing the number of panicle primordia [49]. This reveals a growth–defense trade-off mechanism mediated by ABA in the allocation of nitrogen between vegetative growth and reproductive organs. Sun et al. 2020 [50] demonstrated that increased external nitrate or ammonium concentrations induce rapid accumulation of abscisic acid (ABA) in both shoots and roots. Sun et al. [51] found that ABA alleviates ammonium toxicity by activating the antioxidant system and enhancing the GS/GOGAT cycle, thereby reducing reactive oxygen species (ROS) and free ammonium accumulation. This process is mediated by the ABA-responsive *OsSAPK9–OsbZIP20* signaling module, in which *OsSAPK9* phosphorylates *OsbZIP20* to induce the expression of downstream protective genes. Knockout mutants of either gene lose ABA-induced ammonium tolerance, highlighting the specific regulatory role of this pathway under high NH_4_^+^ stress [51]. Liu et al. [52] demonstrated that low doses of exogenous ABA can alleviate acid rain damage and promote nitrate uptake in rice, further indicating the adaptive regulatory role of ABA in nutrient acquisition under environmental stress. Hang et al. [53] found that *OsNPF3.1*, a key gene for nitrate and hormone transport, plays an important regulatory role in rice tillering and NUE. Overexpression of *OsNPF3.1* significantly increased ABA accumulation in roots and GA levels at the plant base, which inhibited the germination of rice tiller buds (especially under high nitrate) and thereby reduced the number of tillers. Under low- and moderate-nitrate conditions, *OsNPF3.1* overexpression significantly improved plant NUE [53]. Xu et al. (2024) [54] recently discovered that abscisic acid (ABA) not only responds to nitrogen stress but also integrates nitrate signaling through crosstalk with the core *PYR/PYL–PP2C–SnRK2* ABA pathway and NLP7-dependent transcriptional regulation of nitrate-responsive genes. These findings highlight the nuanced and complex role of ABA in coordinating nitrogen sensing, root system architecture, and stress resilience. In summary, ABA can significantly enhance nitrogen uptake and utilization efficiency by regulating root development, the expression of nitrogen transporter proteins, the activity of nitrogen metabolism enzymes, and nitrogen allocation. During crop growth, ABA concentration and signaling activity may be adjusted in response to nitrogen availability, helping plants optimize nitrogen uptake and utilization under varying environmental conditions. Applying ABA or enhancing ABA signaling pathways through genetic engineering can significantly improve crop NUE, especially under nitrogen-deficient conditions.

### 2.4. Ethylene

Ethylene is a gaseous plant hormone involved in multiple physiological processes, playing important roles in seed germination, root hair development, organ senescence, and fruit ripening (Table 1). Ethylene is synthesized via the Yang cycle, which begins with the conversion of methionine into S-adenosyl-L-methionine (SAM). SAM is then converted into 1-aminocyclopropane-1-carboxylic acid (ACC) by ACC synthase (ACS), and, finally, ACC is oxidized to ethylene by ACC oxidase (ACO) [55].

In recent years, studies have found that ethylene is becoming more and more important in the mechanism of nitrogen uptake and utilization in rice. Li et al. [56] found that inoculation with an endophytic fungus (strain “Maple”) significantly increased auxin, cytokinin, and ethylene levels in rice under low-nitrogen conditions, while also increasing plant nitrogen accumulation and yield and inducing the expression of genes related to nitrogen uptake and metabolism. In this study, exogenous application of auxin, cytokinin, or ethylene enhanced nitrogen accumulation in rice, whereas applying their inhibitors reduced nitrogen uptake. This indicates that ethylene plays an important regulatory role. Ethylene regulates the expression of *OsPAD4* and *OsNRT2* genes, orchestrating the formation of aerenchyma and thereby reshaping root architecture to influence nitrogen acquisition [57]. Li et al. [58] confirmed in field studies under water-saving irrigation conditions that ethylene-mediated root plasticity enhances rice root surface area and nitrogen use efficiency by promoting the formation of aerenchyma. In addition, ethylene may act as an interplant signaling mediator under high-planting-density stress. Studies have shown that nitrogen availability affects rice growth in an ethylene-dependent manner, indicating that regulating ethylene homeostasis is crucial for rice growth under dense planting conditions [59]. Under both nitrogen-deficient (3 mM NO_3_^−^) and nitrogen-sufficient (10 mM NO_3_^−^) conditions, high planting density negatively affects rice growth. However, ethylene promotes root growth by increasing root hair number and density, thereby expanding the absorptive surface area of the root system [59]. Under nitrogen deficiency and drought stress conditions (induced by PEG), upregulation of the *OsACS5* gene enhances ethylene biosynthesis, which stimulates root hair elongation and lateral root initiation by synergistically activating ethylene and auxin-responsive genes [60]. Ethylene treatment significantly upregulates the expression of nitrate and ammonium transporter genes in seedlings [61], confirming its direct role in enhancing nitrogen use efficiency (NUE). Notably, nitrate (NO_3_^−^) induces ethylene synthesis more efficiently than ammonium (NH_4_^+^), whereas ammonium enhances water uptake by stimulating aquaporin expression independent of ethylene biosynthesis, reflecting a nutrient-specific hormonal response mechanism. Ethylene is also involved in the ammonium tolerance process of rice, a naturally ammonium-tolerant species: high concentrations of ammonium (15 mM NH_4_^+^) significantly induce the expression of ethylene biosynthesis and signaling genes such as *OsETO1*, *OsERS1*, *OsEIL2*, and *OsERF83* [62]. However, excessive ethylene may be harmful: recent mechanistic studies by Wang et al. [63] indicate that under soil compaction or hypoxic conditions, ethylene accumulation may inhibit root elongation due to excessive formation of aerenchyma and reactive oxygen species (ROS) accumulation.

In summary, ethylene directly or indirectly affects rice NUE by regulating processes such as nitrogen uptake, transport, and distribution in roots. Future research on ethylene’s role in rice nitrogen uptake and utilization should focus on detailed molecular mechanisms, especially how ethylene interacts with nitrogen signaling pathways under different nitrogen sources and regimes. Currently, most studies on nitrogen–ethylene interactions use simple and controlled nitrogen conditions, whereas in natural environments nitrogen sources are more complex and dynamic. Therefore, investigating ethylene’s regulatory role under more realistic field conditions will be a priority. Furthermore, the interaction mechanisms between ethylene and other phytohormones (auxin, cytokinin, gibberellin, etc.) remain underexplored. By examining the synergistic effects of ethylene with other phytohormones, new insights into improving rice NUE may be gained. Moreover, with advancements in molecular biology, emerging techniques like ChIP-seq and ATAC-seq will help pinpoint key genes and regulatory networks through which ethylene controls nitrogen uptake. Such studies will enhance our overall understanding of rice nitrogen uptake mechanisms and provide theoretical support for improving crop NUE, reducing excessive fertilizer use, and promoting sustainable agriculture.

### 2.5. Other Phytohormones

Gibberellins (GAs) (Table 1) are synthesized via the terpenoid pathway. Geranylgeranyl diphosphate (GGDP) is first converted into ent-copalyl diphosphate (ent-CDP) by ent-copalyl diphosphate synthase (CPS), followed by its transformation into ent-kaurene by ent-kaurene synthase (KS). Subsequently, ent-kaurene is oxidized by cytochrome P450 monooxygenases, including ent-kaurene oxidase (KO) and ent-kaurenoic acid oxidase (KAO), to produce GA_12_, which serves as a common precursor for multiple bioactive GAs [64,65]. GAs play vital roles in regulating plant growth and development. GA promotes vegetative and reproductive growth by triggering the degradation of DELLA proteins, which are major growth repressors in GA signaling. GA is especially important for optimizing crop growth under high-nitrogen conditions, where it helps improve plant architecture and increase yield. Plant responses to GA are part of a complex regulatory network that provides spatiotemporal specificity of gene expression; by integrating GA signaling, plants can coordinate responses to multiple internal signals and environmental factors. This network dynamically links developmental processes with external environmental changes through precise gene regulation. It has been reported that increasing the level of rice Growth-Regulating Factor 4 (GRF4) promotes plant growth, carbon fixation, and nitrogen assimilation, counteracting the inhibitory effect of DELLA proteins [66]. GRF4 directly binds to and activates the expression of genes related to nitrogen assimilation (e.g., *OsGS2* and *OsNR2*), carbon fixation (e.g., *OsLhca1* and *OsTPS1*), and cell proliferation (e.g., *OsCycA1;1* and *OsCdc20s-3*), indicating that GRF4 is a key regulator of growth and metabolic homeostasis [66]. In a recent review, Xing et al. proposed that modulation of the GRF4–DELLA–NGR5 module coordinates nitrogen signaling with hormone pathways to enhance nitrogen use efficiency in rice [67]. However, field trials have shown that excessive accumulation of gibberellins (GAs) can sometimes cause overly vigorous vegetative growth at the expense of grain yield, highlighting the importance of balanced hormone regulation [67]. In conclusion, manipulating the GRF4–DELLA–NGR5 module provides a straightforward strategy for improving NUE by coordinating plant growth and metabolism.

Brassinosteroids (BRs) (Table 1): BRs were initially isolated from rapeseed (Brassica napus) pollen due to their ability to promote cell elongation. Brassinosteroids (BRs) are synthesized from a C28 sterol precursor, campesterol, via the mevalonate (MVA) pathway. This process begins with the reduction of campesterol to campestanol by the 5α-reductase enzyme DET2. Subsequently, a series of cytochrome P450 enzymes mediate sequential hydroxylation and oxidation steps: DWF4 (CYP90B1) catalyzes the rate-limiting C-22 hydroxylation; CPD (CYP90A1) mediates C-23 hydroxylation; ROT3/CYP90D1 is involved in C-2/C-3 epoxidation; and BR6ox (CYP85A1/A2) catalyzes C-6 oxidation. These reactions ultimately lead to the formation of the bioactive BRs, castasterone and its lactonized derivative brassinolide [68]. Brassinolide (the most active BR) plays a significant role in regulating nitrogen uptake and metabolism, thereby enhancing NUE and improving crop yield and quality. It has been reported that BR treatment can effectively increase nitrate and ammonium accumulation in both shoots and roots [69]. NH_4_^+^ promotes the biosynthesis of BR in rice by upregulating the transcript of BR-deficient dwarf (*OsBRD1*), which is directly regulated by five MADS-box transcriptional repressors, thus influencing root growth [70]. The expression of the BR signaling transcription factor BEP1 is significantly induced under nitrogen deficiency, and BEP1 directly binds to the promoters of *NRT2.1* and *NRT2.2*, promoting their expression and enhancing nitrate uptake under nitrate-deficient conditions [71]. Yang et al. [69] demonstrated in a field study that foliar application of brassinosteroids (BRs) enhances nitrogen use efficiency and yield in rice, likely through coordinated upregulation of nitrate transporters and improved root architecture.

Strigolactones (SLs) (Table 1): SLs were first identified as rhizosphere signaling molecules that stimulate the germination of parasitic weeds and branching of arbuscular mycorrhizal fungi [53]. Strigolactones (SLs) are synthesized from β-carotene, which is first isomerized by DWARF27 (D27). The isomerized intermediate is then sequentially cleaved by two carotenoid cleavage dioxygenases, CCD7 and CCD8, to produce carlactone, a central biosynthetic precursor. Carlactone is further oxidized by cytochrome P450 enzymes such as MORE AXILLARY GROWTH1 (MAX1) to generate diverse bioactive SLs. In rice, *OsD27*, D17 (homolog of CCD7), D10 (homolog of CCD8), and MAX1 homologs are key genes involved in SL biosynthesis [72,73]. Since then, SLs have been recognized as phytohormones that regulate shoot branching and root development. They are involved in plants’ adaptive responses to variable soil nutrient supplies—for example, promoting root elongation under low-nitrogen conditions to explore a greater soil volume for nitrogen [74]. Luo et al. (2018) [75] found that exogenous application of the synthetic SL analog GR24 altered nitrogen allocation in the leaves of an SL-deficient mutant (d10), while having no significant effect on an SL signaling mutant (d3) or on the wild type. SLs can influence plant growth by regulating the partitioning of nitrogen to different above-ground tissues. When rice roots experience nitrogen deficiency, the SL signaling pathway positively regulates NUE, promoting nitrogen uptake. Advances in understanding the coordinated regulation of metabolism and development by GA and SL signaling have provided new insights for breeding strategies to improve nitrogen efficiency, contributing to a sustainable “green revolution” in rice production.

### 2.6. Multi-Hormone Synergistic Network and Systemic Regulation

The optimization of nitrogen use efficiency (NUE) in rice depends on a dynamic and synergistic network composed of multiple plant hormones. This network achieves system-level integration of nitrogen signal perception, developmental responses, and environmental adaptation through cascade signal transduction, transcriptional regulatory crosstalk, and metabolic feedback (Table 2, Table 3, Table 4 and Table 5). 

## 3. Conclusions and Perspectives

**Figure 1:**
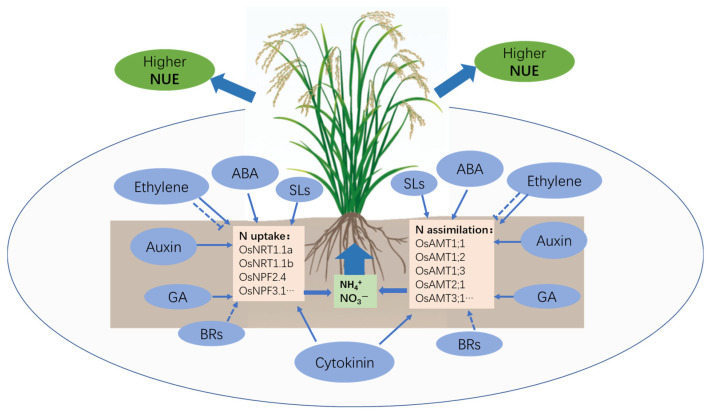
Schematic representation of the effects of phytohormones on nitrogen use efficiency in rice. The blue ellipse represents the synthesis and signal transduction pathways of phytohormones. Solid-lined arrows represent activation of gene expression. Bar-headed dotted lines represent partial inhibition of gene expression. Dotted-line arrows denote a positive regulation by different mechanisms not entirely characterized.

In summary, plant hormones have emerged as central regulators of nitrogen use efficiency (NUE) in rice, integrating multiple pathways governing growth, development, and stress responses (Figure 1). Collectively, these hormone-driven mechanisms enable rice to maintain high yields under varying nitrogen conditions, establishing a robust physiological foundation for sustainable green revolution practices. Rice experiences different growth environments during its growth and development, and phytohormones are of great significance for improving its nitrogen use efficiency. This review found that auxin, cytokinin, ABA, GA, ethylene, BRs, and SLs can improve NUE at different growth and development stages of rice, which provides theoretical support for exogenous spraying of phytohormones to regulate NUE and also lays a theoretical foundation for the development of new plant growth regulators.

Although progress has been made in understanding how phytohormones regulate crop NUE, many challenges and unknowns remain. As agriculture increasingly demands environmentally friendly technologies and efficient resource use, in-depth research into the mechanisms and application potential of phytohormones for improving crop NUE has become a key direction for future agricultural innovation. In-depth investigation of the regulatory mechanisms of phytohormones on nitrogen use efficiency (NUE) using molecular tools such as genomics, transcriptomics, and gene editing will lay the foundation for elucidating the key steps in nitrogen uptake and assimilation [67,76,79]. Plant responses to nitrogen use under stress conditions often rely on the regulation of complex phytohormone networks [54,77,80]; in this regard, research and exploration need to be strengthened. It is necessary to introduce hormone-related regulatory factors identified in the laboratory into test cultivars, and to validate their effects on NUE under diverse environmental conditions through the integration of precision fertilization, field monitoring, and multi-location trials.

## Figures and Tables

**Table 1:** Summary of phytohormones, their functions, and effects on nitrogen uptake.

Hormone	Year of Discovery	Key Plant Parts	Effect on Nitrogen Uptake	Molecular Structure
Auxin (IAA)	1930s	Roots, stems, leaves, flowers, seeds	Enhances nitrogen uptake under low-nitrogen conditions, affects ammonium utilization	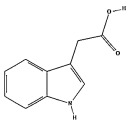
Cytokinin	1950s	Roots, shoots, leaves	Increases nitrogen use efficiency by regulating nitrogen distribution and metabolism	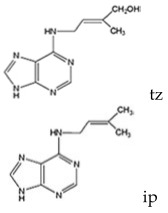
ABA	1960s	Roots, leaves, seeds	Enhances nitrogen uptake by regulating root development and nitrogen transporter expression	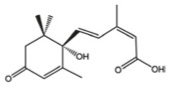
Ethylene	Early 1900s	Roots, shoots, leaves	Promotes root growth and increases nitrogen uptake under high planting density and nitrogen deficiency	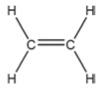
Gibberellins	1920s	Stem tip, young leaves, immature seeds, root system	Enhance nitrogen accumulation under sub-low temperature conditions and promote energy and carbon skeleton supply	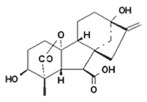
Brassinosteroids	1970s	Pollen, seeds, fruits, stem tips, young internodes, roots	Regulates nitrogen transport genes in collaboration with other hormones (such as auxin)	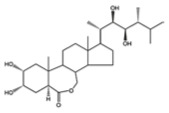
Strigolactones	2000s	Root exudates, root tips, vascular tissue	Indirect inhibition (reducing nitrogen demand through branching regulation) or promoting mycorrhizal symbiosis to increase nitrogen	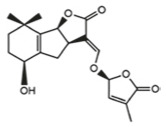

**Table 2:** Nitrogen signal perception and root synergy.

Hormone Combination	Synergistic Mechanism	Functional Output	References
IAA-CTK-SLs Triangular Hub	Nitrate activates IAA signaling via *OsNRT1.1A* → Induces *OsIPT* expression to promote CTK synthesis → SLs suppress lateral branching through D14-SMAX1 module	Dynamically balances tillering and nitrogen acquisition efficiency	[15,33,37,74,75]
ABA-ETH-BR Antioxidant Axis	ABA-activated *OsSAPK9* phosphorylates bZIP20 + ETH-induced *OsEIL2* + BR-stabilized BES1 → Synergistically upregulate *OsGS1;2* and *SOD* genes	Alleviates ammonium toxicity and ROS accumulation	[51,61,62,69]
IAA-ETH Aerenchyma Module	Hypoxia-induced ETH accumulation → Inhibits PIN2-mediated IAA efflux → IAA accumulation at root tips triggers programmed cell death in cortex	Forms aerenchyma to enhance low-N adaptability	[29,55,57,60]

Notes: Arrows indicate dynamic changes or regulatory relationships: ↑ increase/upregulation; ↓ decrease/downregulation; → induction/activation. Example: ABA↑ denotes elevated abscisic acid levels; IAA→PIN2 indicates auxin activates PIN2 expression.

**Table 3:** Tillering and source–sink regulation.

Regulatory Pathway	Molecular Interactions	Physiological Effect	References
CTK-BR-NGR5 Tillering Promotion	BR-induced BES1 binds CTK-responsive factor RR21 → Activates *OsNRT2.1* GA degrades DELLA to release NGR5 → Recruits PRC2 to repress tillering gene *FC1*	Increases panicle number by 19–30% under sufficient N	[45,66,69,71,76]
ABA-GA Sink Strength Allocation Axis	ABA phosphorylates DELLA via SnRK2 during grain filling → Releases GRF4 suppression → Activates grain N transporter *OsGluA2*	Enhances nitrogen harvest index by 15–20%	[49,54,66,67]
SLs-IAA N-P Balance	Under low P: SLs suppress BR synthesis gene *D11* → Reduces lateral roots Under low N: IAA inhibits *D27* → Decreases SLs biosynthesis	Optimizes N-P allocation ratio	[70,72,73,74]

Note: Symbols same as Table 2.

**Table 4:** Environmental adaptation and stress response.

Stress Type	Hormonal Dynamics	Synergistic Response Mechanism	Agronomic Significance
High-Density Planting	ETH↑, BR↓, ABA↑	ETH suppresses BR signaling → Reduces plant height ABA closes stomata → Decreases transpiration SLs inhibit tillering → Concentrates N for panicles	Increases population N efficiency by 12–18% [59,69,77]
Drought–Low N Co-stress	ABA↑, CTK↓, IAA↓	ABA activates *OsPP2C09* to inhibit ammonium uptake Downregulates CTK synthesis gene *OsIPT4* Induces IAA oxidase to reduce auxin accumulation	Water conservation and nitrogen retention [42,52,54]
Ammonium Toxicity	ABA↑, BR↑, ETH→	ABA-BR activates antioxidant enzymes via OsCIPK18-bZIP20 ETH enhances AMT1;3 phosphorylation to reduce NH_4_^+^ uptake	Reduces biomass loss by 40% [51,62,71]

Note: Symbols same as Table 2.

**Table 5:** Mycorrhizal symbiosis and systemic signaling.

Symbiosis Type	Hormonal Action	Nitrogen Acquisition Pathway	References
SLs-AMF Organic N Network	Low N induces SLs secretion → Activates fungal CCD1 branching factors → Hyphae expand rhizosphere scope → Hyphae transport amino acids/peptides	Organic N uptake increased by 18–24%	[72,75,78]
IAA-ACC Microbial Interaction	Rhizospheric ACC signals promote diazotroph colonization → Microbially synthesized IAA feedback enhances root growth → Activates AMT1 ammonium transporters	Biological N fixation contribution increased by 15%	[19,56,58]

Note: Symbols same as Table 2.
